# Efficient and Stable Photocatalytic Hydrogen Evolution Activity of Multi-Heterojunction Composite Photocatalysts: CdS and NiS_2_ Co-modified NaNbO_3_ Nanocubes

**DOI:** 10.3389/fchem.2019.00880

**Published:** 2020-01-21

**Authors:** Jingjing Xu, Jiawei Zhu, Junfeng Niu, Mindong Chen, Junpeng Yue

**Affiliations:** ^1^Jiangsu Key Laboratory of Atmospheric Environment Monitoring and Pollution Control, Collaborative Innovation Center of Atmospheric Environment and Equipment Technology, Jiangsu Engineering Technology Research Center of Environmental Cleaning Materials, School of Environmental Science and Engineering, Nanjing University of Information Science and Technology, Nanjing, China; ^2^Research Center for Eco-Environmental Engineering, Dongguan University of Technology, Dongguan, China

**Keywords:** H_2_ evolution, noble-metal-free cocatalyst, NaNbO_3_, CdS, NiS_2_

## Abstract

In this study, a NaNbO_3_/CdS/NiS_2_ ternary composite photocatalyst containing no precious metals was successfully prepared by a simple hydrothermal method. The prepared ternary photocatalyst has a significant improvement in photocatalytic performance of hydrogen production from water splitting under visible light irradiation. The best sample NCN40% hydrogen production rate is 4.698 mmol g^−1^ h^−1^, which is about 24.7 times that of pure CdS sample. In addition, the stability of the composite catalyst in the long-term photocatalytic hydrogen production cycle is also improved. The reason for the enhanced hydrogen production performance may be the optimization of the microstructure of the catalyst and the reduction of photogenerated electron-hole recombination. The construction of multi-heterojunctions (NaNbO_3_-CdS, CdS–NiS_2_, and NaNbO_3_-NiS_2_) helps to reduce the recombination of carriers. Furthermore, the *in-situ*-formed NiS_2_ nanoparticles can serve as active sites for hydrogen evolution. All of these factors induced the improved photocatalytic activity of the as-prepared ternary photocatalyst.

## Introduction

Hydrogen is regarded as the clean energy with the greatest development potential in the twenty-first century, with the advantages of high calorific value and no pollution (Esswein and Nocera, 2007; Dempsey et al., 2009; Wang et al., 2018; Mu et al., 2020a). It is a fact that 70% of the earth's surface is water, so many researchers focus on obtaining hydrogen energy through photolysis of water (Hou et al., 2017; Ruan et al., 2018; Yan et al., 2018; Zhang C. et al., 2018; Yu et al., 2019). Many semiconductor-based photocatalysts such as sulfides, nitrides, and metal oxides have been synthesized and studied for photocatalytic hydrogen production (Yu et al., 2016; Zhang G. et al., 2017; Wu et al., 2018; Li J. et al., 2019; She et al., 2019). However, the performance of these photocatalysts is still unsatisfactory, so more new materials need to be developed.

NaNbO_3_ is a photocatalyst with a typical perovskite structure and also has excellent chemical stability, ionic conductivity, and photocatalytic properties (Li et al., 2009; Kim et al., 2013; Liu et al., 2017; Sun et al., 2017; Zhang B. et al., 2017; Chen et al., 2018). Although, NaNbO_3_ has the advantages of redox ability due to large band gap, it also has disadvantages like low charge separation efficiency and low photocatalytic activity (Xu et al., 2015). There have been many reports that NaNbO_3_ was combined with C_3_N_4_, Bi_2_O_3_, CeO_2_, etc. to ameliorate optical performance and limit recombination of charge carriers (Shi et al., 2009; Chen et al., 2014; Qian et al., 2018; Qiao et al., 2019; Singh Vig et al., 2019; Yang F. et al., 2019).

Cadmium sulfide has been studied a lot, which is attributed to the suitable forbidden band width and conduction band position (Cao et al., 2013; Lang et al., 2016; Li et al., 2017; Ma et al., 2017). Although, the narrower bandwidth has higher spectral utilization efficiency, it also brings the disadvantages of low electron–hole separation efficiency and easy corrosion by light (Li et al., 2011; Zhou et al., 2017; Ruan et al., 2018; Dong et al., 2019; Wang et al., 2019a). Dispersing CdS on other nanomaterials and constructing heterojunctions have been shown to improve the photocatalytic activity and reduce photo-etching (Ke et al., 2019; Xu et al., 2019; Yue et al., 2019). According to the excellent stability of NaNbO_3_ and the shortcomings of CdS photocatalyst, dispersing CdS on the surface of NaNbO_3_ may be a method to increase photocatalytic activity. Active sites are critical to increasing the yield of water splitting. For example, there have been many reports on the preparation of precious metals cocatalyst-modified photocatalysts that can help in restricting the recombination of electrons and holes (Cao et al., 2015; Huang et al., 2017; Naskar et al., 2017; Zhang Y. et al., 2018; Liu et al., 2019; Yang X. et al., 2019). Precious metals are expensive and have very low reserves, so it is necessary to look for the same highly efficient non-precious metals as a substitute for precious metals (Xu and Xu, 2015; Xing et al., 2017; Kang et al., 2019; Wang et al., 2019b; Chen et al., 2020). It has been reported that a compound of a transition metal Ni was used as a cocatalyst to greatly enhance photocatalytic activity (Chen et al., 2017, 2019; Ma et al., 2017; Digraskar et al., 2019; Li H. et al., 2019; Dong et al., 2020). Therefore, it is possible to introduce NiS_2_ into theNaNbO_3_/CdS system to synthesize ternary composites, further facilitating the separation of charge carries and increasing hydrogen evolution rate.

In this paper, we designed and synthesized a noble-metal-free ternary composite NaNbO_3_/CdS/NiS_2_ (NCN) in which NaNbO_3_ was used as a carrier to uniformly disperse CdS and NiS_2_. Multi-heterojunctions (NaNbO_3_-CdS, CdS–NiS_2_, and NaNbO_3_-NiS_2_) were constructed and NiS_2_ was used as cocatalyst, enriching photogenerated electrons to improve the kinetic of hydrogen evolution. The as-synthesized NCN40% sample had a photocatalytic hydrogen evolution rate of 4.699 mmol g^−1^ h^−1^, which had great improvement compared to pure CdS. NaNbO_3_ itself has excellent electrical conductivity and chemical stability. It not only acts as a carrier for CdS to increase the reaction surface with water but also forms a heterojunction with CdS, which can improve light irradiation stability and accelerate charge separation (Al Balushi et al., 2018). As an active site, NiS_2_ can improve photocatalytic activity and facilitate the separation of electron–hole pairs.

## Experimental

### Synthesis of NaNbO_3_ Nanocubes and NaNbO_3_/CdS/NiS_2_ Samples

The NaNbO_3_ cubes were synthesized by a hydrothermal method (Qian et al., 2018). A total of 2.0 g of Nb_2_O_5_ was added into sodium hydroxide solution (120 ml, 10 mol/l) and stirred for 120 min. After that, the suspension was heated in a 200-ml Teflon container and kept at 150°C for 48 h. After cooling down, the precipitate was centrifuged and washed with ethanol and pure water for several times until the pH was about 7. Finally, after drying at 60°C, NaNbO_3_ nanocubes were obtained.

*Synthesis of NaNbO*_3_*/CdS/NiS*_2_ *(NCN)*. As synthesized NaNbO_3_ (1.448, 0.7224, and 0.4816 g) was put into pure water (150 ml) and ultrasonically processed for 30 min to disperse entirely. Then, Cd(CH_3_COO)_2_·2H_2_O (2 mmol), CH_4_N_2_S (4 mmol), and Ni(NO_3_)_3_·6H_2_O (0.2 mmol) were added, and the suspension was stirred for 0.5 h. Afterwards, the suspension was heated at 160°C for 12 h. After cooling, the precipitate was centrifuged, washed with pure water and ethanol, and then dried at 60°C. The obtained samples were tabbed as NCN20%, NCN40%, and NCN60%, and the mass ratios of CdS and NaNbO_3_ were 20, 40, and 60%, respectively.

CdS was synthesized as the abovementioned process without addition of NaNbO_3_ and Ni(NO_3_)_3_·6H_2_O. CdS/NiS_2_ and NaNbO_3_/CdS-40% (NC40%) were also prepared in a similar way. Cd(CH_3_COO)_2_·2H_2_O (2 mmol), CH_4_N_2_S (4 mmol), and Ni(NO_3_)_3_·6H_2_O (0.2 mmol) were added into pure water and stirred for 0.5 h. Afterwards, the suspension was heated at 160°C for 12 h. After cooling, the precipitate was centrifuged, washed with pure water and ethanol, and then dried at 60°C. The obtained samples were defined as CdS/NiS_2_. For the preparation of NaNbO_3_/CdS-40% (NC40%), NaNbO_3_ (0.7224 g) was added into pure water (150 ml) and ultrasonically treated for 30 min to disperse entirely. Then, Cd(CH_3_COO)_2_·2H_2_O (2 mmol) and CH_4_N_2_S (4 mmol) were added, and the suspension was stirred for 0.5 h. Afterwards, the suspension was heated at 160°C for 12 h. After cooling, the precipitate was centrifuged and washed with pure water and ethanol before it was dried at 60°C.

### Characterizations

The crystal structure and phase characteristics were determined by an X-ray diffractometer at the speed of 10° min^−1^ with CuKa radiation (XRD-6100). The chemical composition of the composite sample was determined by multifunctional imaging electron spectrometry (Thermo ESCALAC 250Xi). The microstructure and morphology were obtained by high-resolution projection microscopy (US FEI Tecnai G2 F20). UV–vis DRS spectra was measured on Shimazu UV3600. PL (Photoluminescence) spectra were researched by Hitachi F-700 fluorescence spectrophotometer. The Brunauer–Emmett–Teller (BET) surface area was obtained by Nitrogen (N_2_) adsorption–desorption technique at 77 K on a Quantachrome IQ-2 instrument. Prior to the measurement of N_2_ sorption, the samples were activated at 120°C for about 5 h under vacuum.

The photocurrent response and Mott–Schottky curve were operated on the electrochemical station (Chenhua Instruments, CHI760E), which uses a sample membrane, a platinum plate and Ag/AgCl as electrodes, and 0.5 M Na_2_SO_4_ as the electrolyte. The sample membrane was a tin fluoride (FTO) conductor glass, which was evenly coated with sample of 1 × 1 cm. The visible light source was a 300-W xenon lamp with a 400-nm filter.

### Photocatalytic Hydrogen Evolution Measurement

The photocatalytic H_2_ evolution performance was assessed using a photocatalytic activity evaluation system (CEL-SPH2N-D5), followed with gas chromatography (GC-7920) to detect the amount of hydrogen. A total of 25 mg of the photocatalyst, pure water (50 ml), and lactic acid (4 ml) were added into a glass reactor, and the suspension was ultrasonicated and stirred for 5 min for dispersion. After the reactor was connected to the photocatalytic system, continuous pumping took place for more than 20 min to ensure that the entire pipeline was in a vacuum. The visible light source was a 300-W xenon lamp with a 400-nm filter, and room temperature was maintained at 25°C.

## Results and Discussion

### Characterization

**Figure 1:**
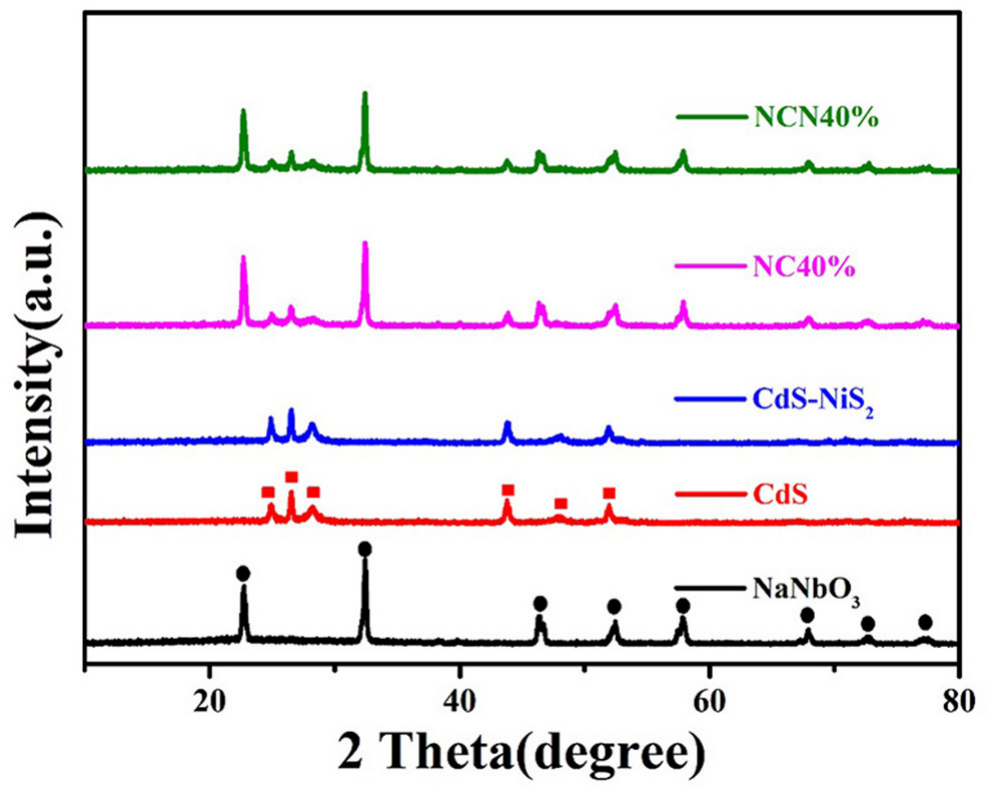
XRD patterns of NaNbO_3_, CdS, CdS-NiS_2_, NC40%, and NCN40%.

Figure 1 compares the XRD spectra of the as-synthesized samples to study the crystal phase. Characteristic diffraction peaks at 22.7°, 32.4°, 46.7°, 52.3°, 57.8°, 67.9°, 72.7°, and 77.2° matched with the diffraction peaks of NaNbO_3_ (Qian et al., 2018). Characteristic diffraction peaks at 24.8°, 26.5°, 28.2°, 43.7°, 47.8°, and 51.8° matched with the diffraction peaks of CdS (Al Balushi et al., 2018). Characteristic diffraction peaks of CdS and NaNbO_3_ were found in NC40% and NCN40% composites simultaneously, indicating the successful preparation of the target samples. No significant characteristic peaks of NiS_2_ were found in the NCN and CdS–NiS_2_ composites, probably attributed to low content. Furthermore, there were no impurity peaks.

**Figure 2:**
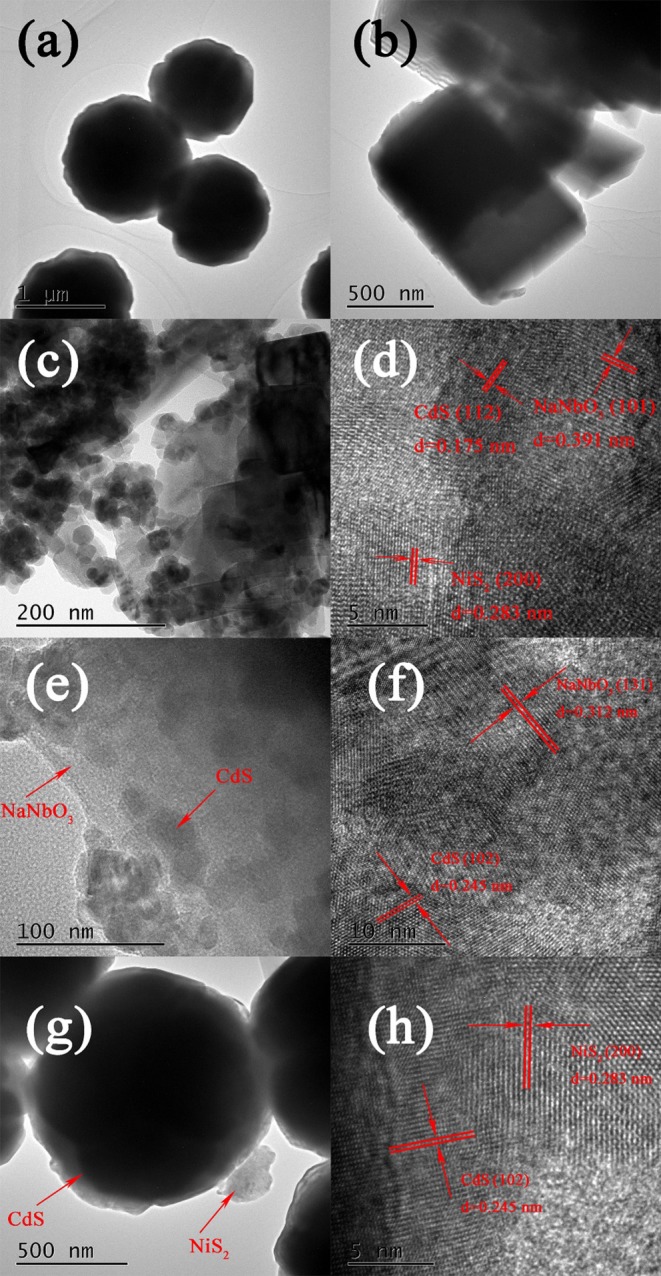
TEM and HRTEM images of **(a)** CdS, **(b)** NaNbO_3_, **(c,d)** NCN40%, **(e,f)** NC40%, and **(g,h)** CdS/NiS_2_.

Figure 2 shows the typical TEM, HRTEM images of NaNbO_3_, and CdS NCN40%. As can be seen from Figures 2a,b, the clustered circular nanoparticles were pure CdS with a diameter of about 1 μm, and the cube shapes were NaNbO_3_ nanocubes. In HRTEM images (Figure 2c), the spherical and cubic species were CdS, NiS_2_, and NaNbO_3_, respectively. CdS and NiS_2_ particles were grown on the NaNbO_3_ cubes uniformly. In Figure 2d, the multi-heterojunctions (NaNbO_3_-CdS, CdS–NiS_2_, NaNbO_3_-NiS_2_) were tightly bound, and NiS_2_ could be observed. The lattice spacing of 0.175, 0.391, and 0.283 nm were observed corresponding to CdS, NaNbO_3_, and NiS_2_, respectively (Ma et al., 2017; Al Balushi et al., 2018; Qian et al., 2018). In addition, Figures 2e–h show the microstructure of NC40% and CdS/NiS_2_. It can be found that the size of CdS in NaNbO_3_/CdS was much smaller than that in CdS/NiS_2_. This proved that the existence of NaNbO_3_ could greatly reduce the agglomeration of CdS.

**Figure 3:**
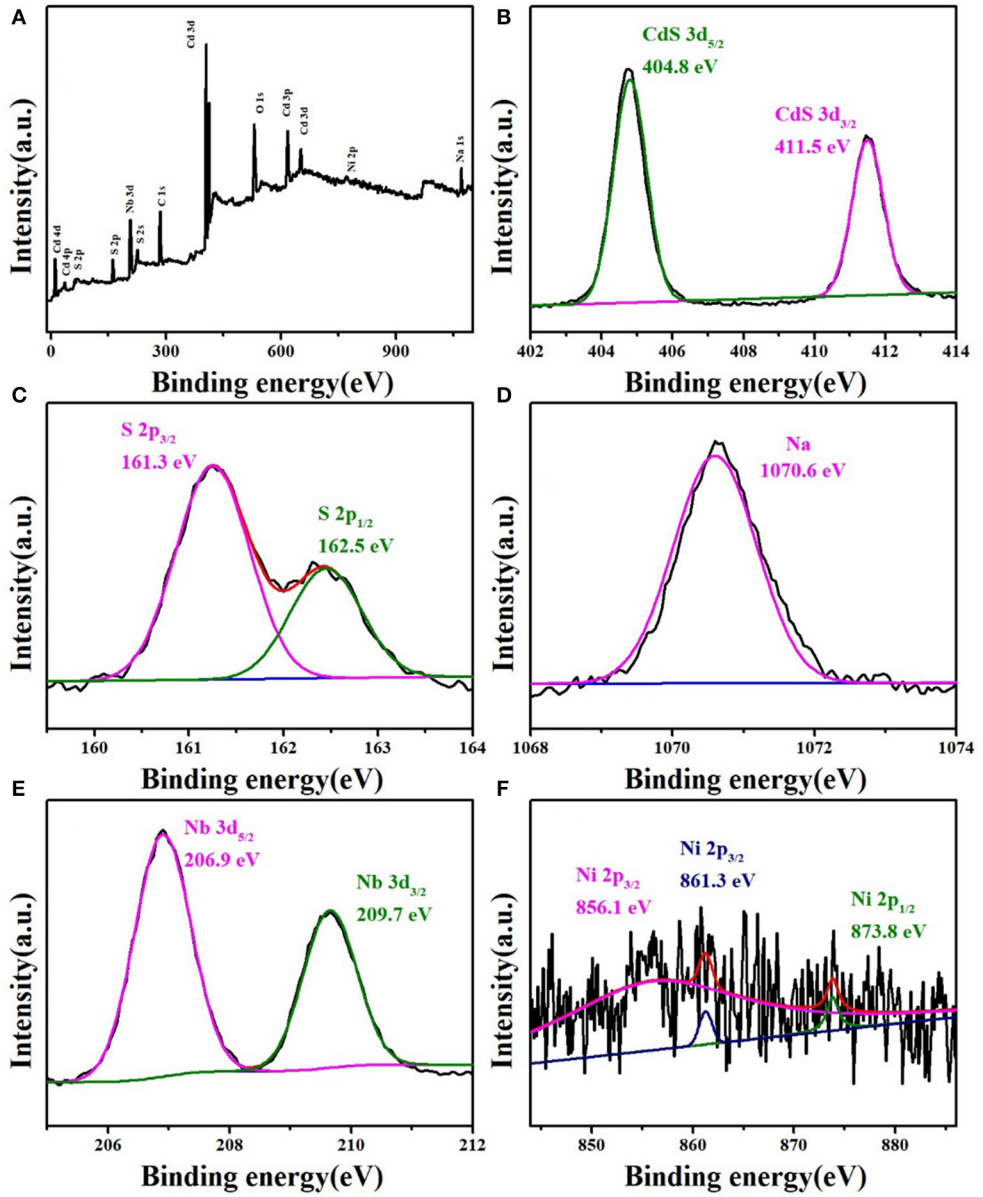
XPS survey spectrum of **(A)** NCN40%. High-resolution XPS spectra of NCN40%: **(B)** Cd 3d, **(C)** S 2p, **(D)** Na, **(E)** Nb 3d, and **(F)** Ni 2p.

Figure 3A shows X-ray photoelectron spectroscopy to confirm the total elemental composition of the NCN40% composite. The Cd, S, Ni, Na, and Nb elements could be found, which further proved the successful preparation of the NCN40% composite sample. The XPS spectra of the elements were also analyzed separately. In Figure 3B, there were characteristic peaks at 404.8 and 411.5 eV (Cd 3d_5/2_ and Cd 3d_3/2_), consistent with past reports (Yu et al., 2015; Ma et al., 2018). S 2p_3/2_ and S 2p_1/2_ were located at 161.3 and 162.5 eV (Figure 3C), corresponding to CdS and NiS_2_ (He et al., 2016; Yue et al., 2019). The peak shown at 1,070.6 eV was Na (Figure 3D) (Wang L. et al., 2017). In Figure 3E, the peaks (206.9 and 209.7 eV) could be attributed to Nb 3d_5/2_ and Nb 3d_3/2_ (Chen et al., 2014). Figure 3F shows two peaks of Ni 2p_1/2_ and Ni 2p_3/2_ at 856.1 and 873.8 eV (Li H. et al., 2019), and the satellite peak of Ni 2p_3/2_ at 861.3 eV (Ma et al., 2017). XPS spectroscopy further proved the successful preparation of the composite sample.

### Photocatalytic H_2_ Production and Mechanism

**Figure 4:**
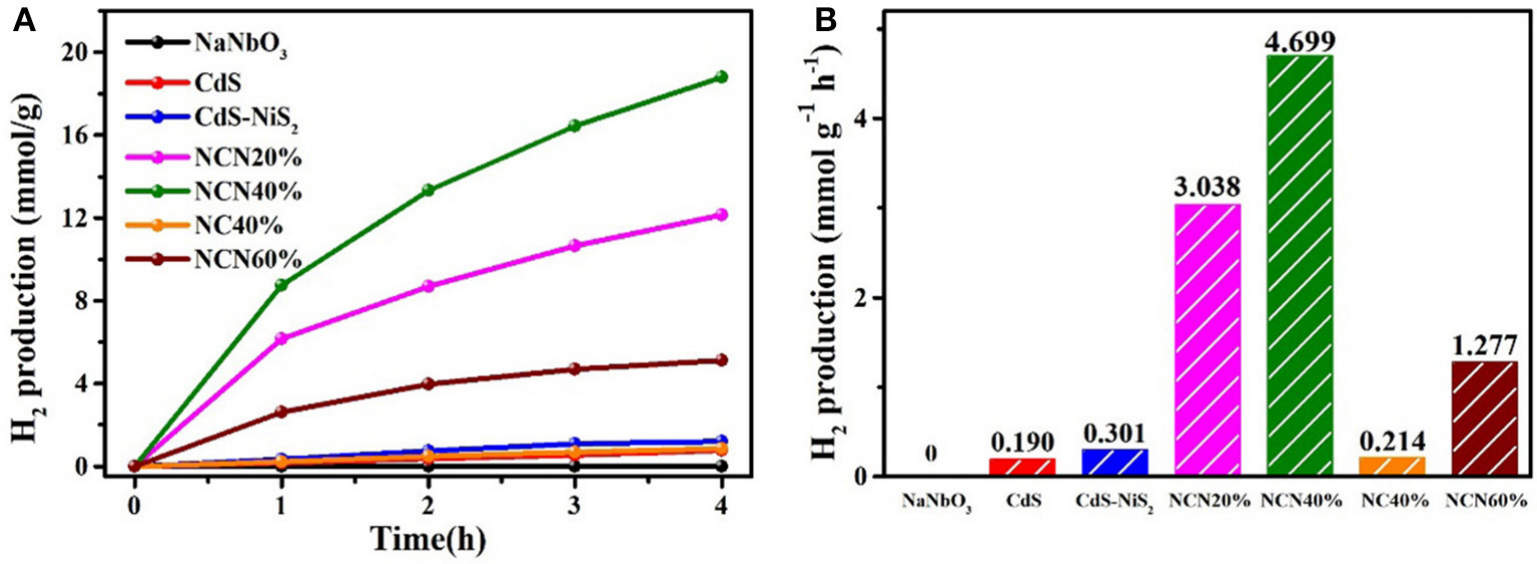
**(A)** H_2_ production amount for various samples vs. irradiation time rate and **(B)** average H_2_ evolution rate under visible light.

Exposed to visible light irradiation, the hydrogen production rates of NCN samples with different NaNbO_3_ contents and CdS, NC40%, and CdS–NiS_2_ were studied (Figures 4A,B). Probably because of a large band gap, pure NaNbO_3_ showed no response to visible light. CdS was responsive to visible light, but H_2_ production efficiency was extremely low (0.190 mmol g^−1^ h^−1^), which may be attributed to the instant reunion of electron–hole pairs. After combining with NiS_2_, the CdS–NiS_2_ sample featured extremely little enhanced hydrogen generation rate (0.301 mmol g^−1^ h^−1^). However, the simultaneous combination with NaNbO_3_ and NiS_2_ greatly boosted the H_2_ evolution activity. Especially, the efficiency of NCN40% composite sample was 4.699 mmol g^−1^ h^−1^, about 24.7 and 21.9 times that of pure CdS and NC40%, respectively. If the content of NaNbO_3_ in the composite sample was low, the CdS particles may not be well-dispersed, and too much NaNbO_3_ may cause light shielding. It indicated that both NaNbO_3_ and NiS_2_ had a great effect on improving photocatalytic efficiency.

**Figure 5:**
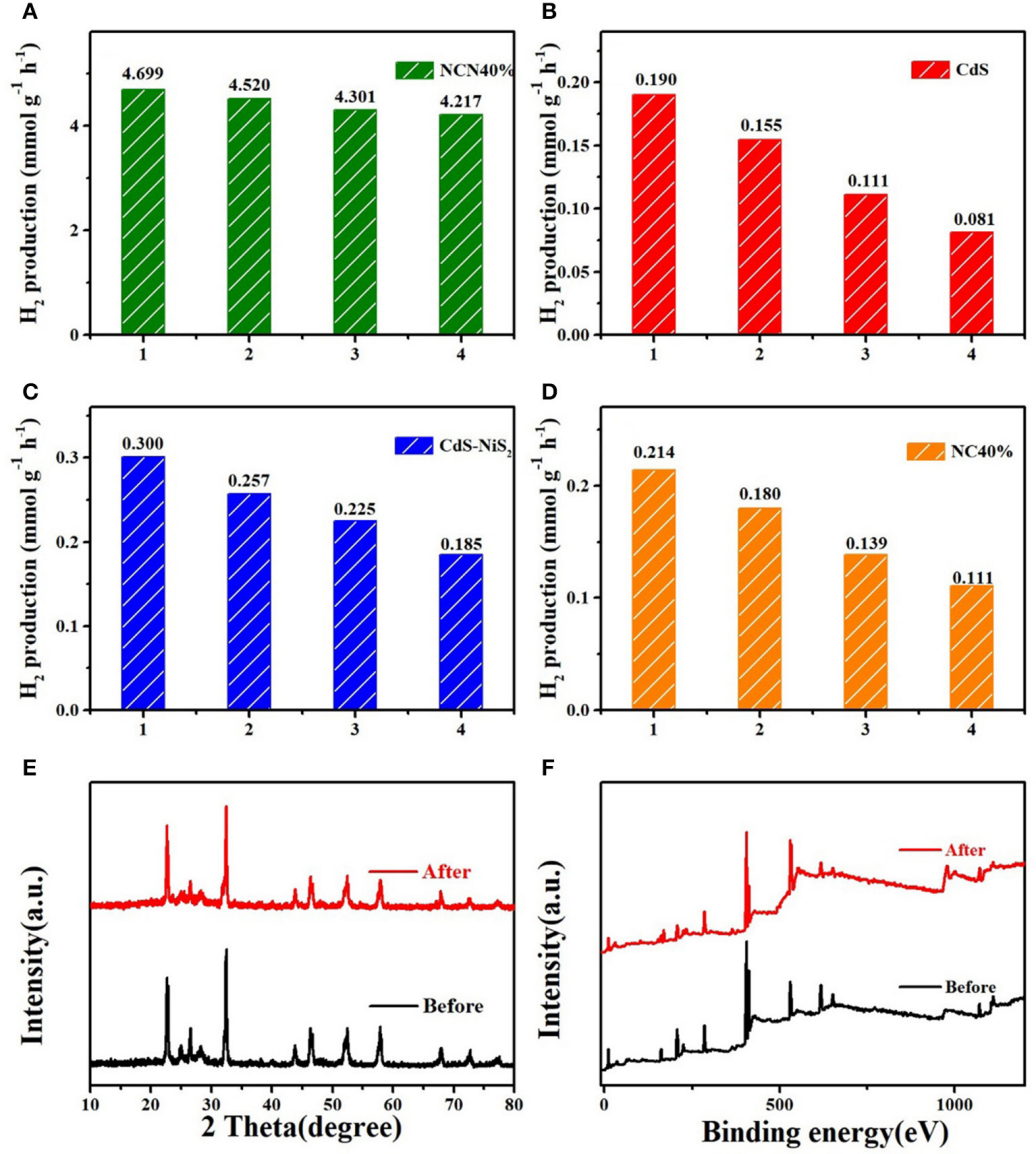
Cycling stability for **(A–D)** NCN40%, CdS, CdS–NiS_2_, and NC40%; **(E)** XRD patterns of NCN40% before and after reaction; **(F)** XPS survey of NCN40% before and after reaction.

At the same conditions, four repeated hydrogen production experiments, each lasting for 4 h, were carried out to evaluate the stability of the abovementioned samples. Figure 5A showed that the NCN40% maintained high hydrogen production efficiency compared to the first cycle, with about 10.3% reduction. Meanwhile, after four repeated tests, the hydrogen evolution efficiency of CdS, CdS–NiS_2_, and CdS–NaNbO_3_ was reduced by 57.4, 38.0, and 48.1%, respectively (Figures 5B–D). In addition, after repeated tests, there was almost no change in the XRD and XPS (Figures 5E,F) spectra of the NCN40% sample compared to the original sample, indicating that the NCN 40% composite had excellent stability under visible light irradiation.

**Figure 6:**
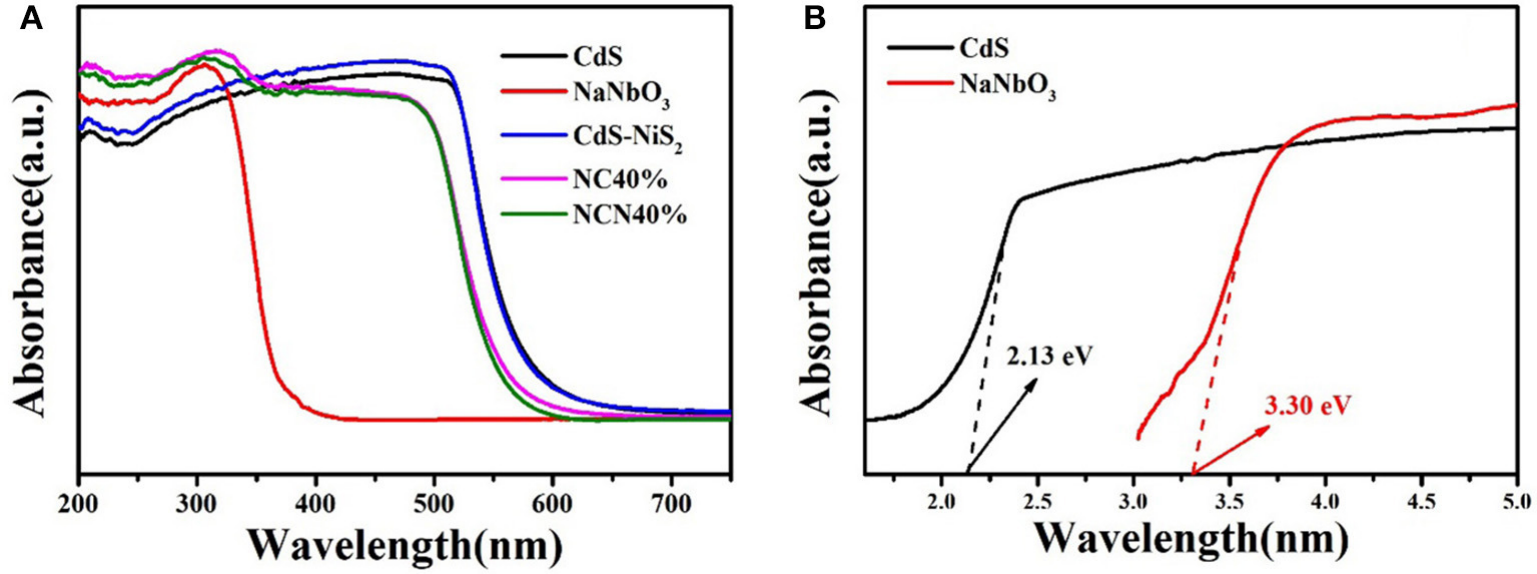
**(A)** Diffuse reflectance spectra for as-synthesized samples and **(B)** Tauc's plot of (α*h*ν)^1/2^ vs. photon energy (*h*ν) of CdS and NaNbO_3_.

DRS patterns (Figure 6) were determined to study the optical properties. Pure NaNbO_3_ and pure CdS showed obvious absorption edge at about 350 and 530 nm, conforming to previous reports, respectively (Xu et al., 2011; Wang Q. et al., 2017). The composite of NiS_2_ had little effect in CdS/NiS_2_ sample. The absorption of the NC40% and NCN40% samples in the ultraviolet region was enhanced, and the light absorption edge was also moved to approximately 500 nm. According to the Kubelka–Munk method: (α*hv*)^1/2^ = *hv*-*E*_g_ (Ren et al., 2019; Mu et al., 2020b), the band gap energy (*E*_g_) of NaNbO_3_ and CdS was 3.30 and 2.13 eV, respectively.

**Figure 7:**
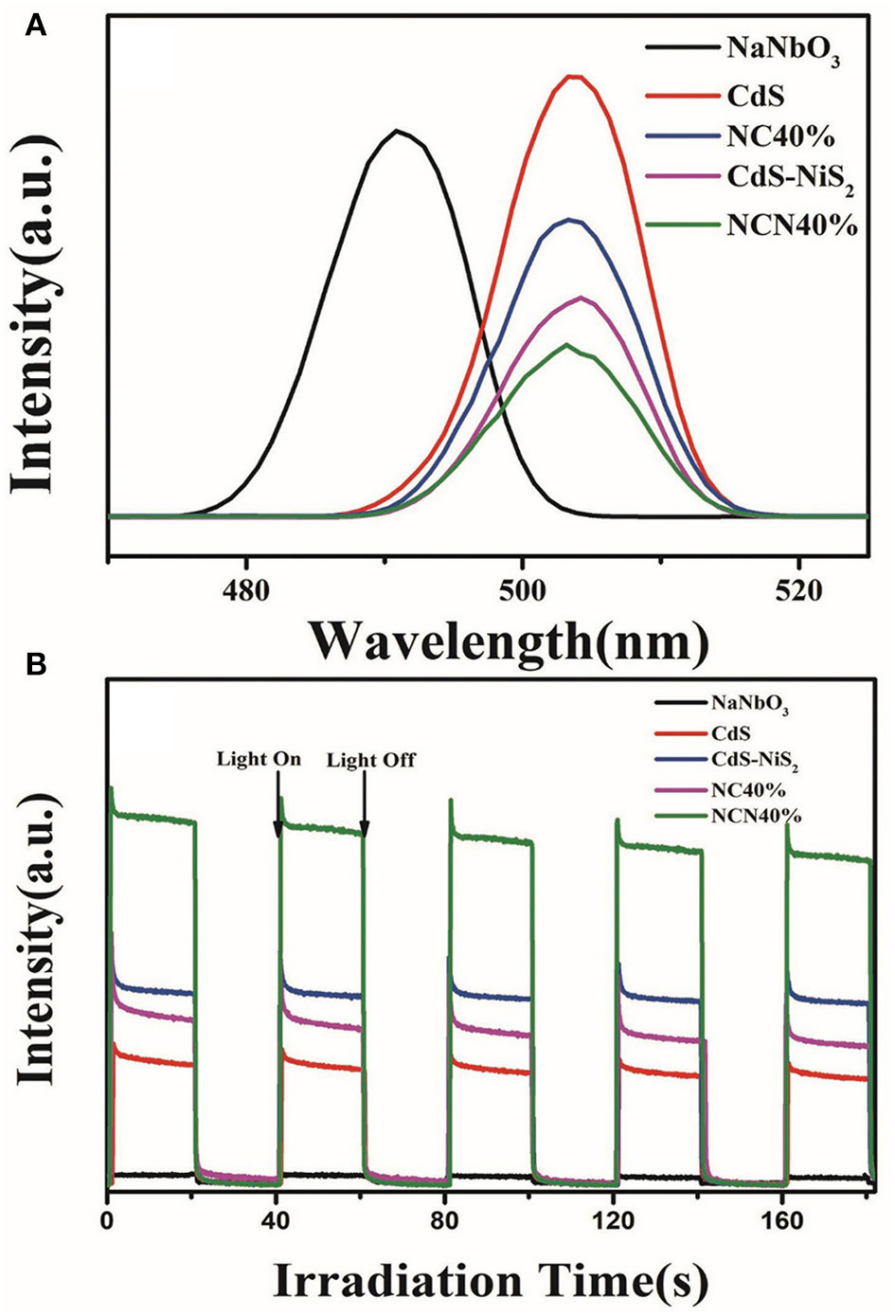
**(A)** Photoluminescence spectra of CdS, NaNbO_3_, CdS-NiS_2_, NC40%, and NCN40%. **(B)** Transient photocurrent response of CdS, NaNbO_3_, CdS-NiS_2_, NC40%, and NCN40% under visible light irradiation.

The photoluminescence test further evaluated the recombination efficiency of electrons and holes. The excitation wavelength was set at 340 nm (Kumar et al., 2014). As shown in Figure 7A, the photoelectrons and holes in the pure CdS were easily recombined, so that the PL emission spectrum was the highest. When CdS was combined with NiS_2_ or NaNbO_3_, the intensity of PL emission spectrum was weakened, indicating that the reunion of charge carriers was suppressed.

Transient photocurrent response was operated and analyzed so as to further study the migration and separation efficiency of charge carriers. In Figure 7B, NaNbO_3_ had almost no photocurrent because it cannot absorb visible light. Then, it was followed by CdS, NC-40%, and CdS-NiS_2_, while the NCN40% composite showed the highest photocurrent intensity. It was consistent with the efficiency of H_2_ evolution rate mentioned above. It indicated that the combination of NaNbO_3_ or NiS_2_ with CdS was beneficial to separate electrons and holes. In NCN composite system, the simultaneous combination of NaNbO_3_, NiS_2_, and CdS, i.e., multi-heterojunction (NaNbO_3_-CdS, CdS–NiS_2_, NaNbO_3_-NiS_2_), was much more significant for the migration efficiency of electrons and holes, meaning that more electrons were used in the hydrogen evolution reaction.

**Figure 8:**
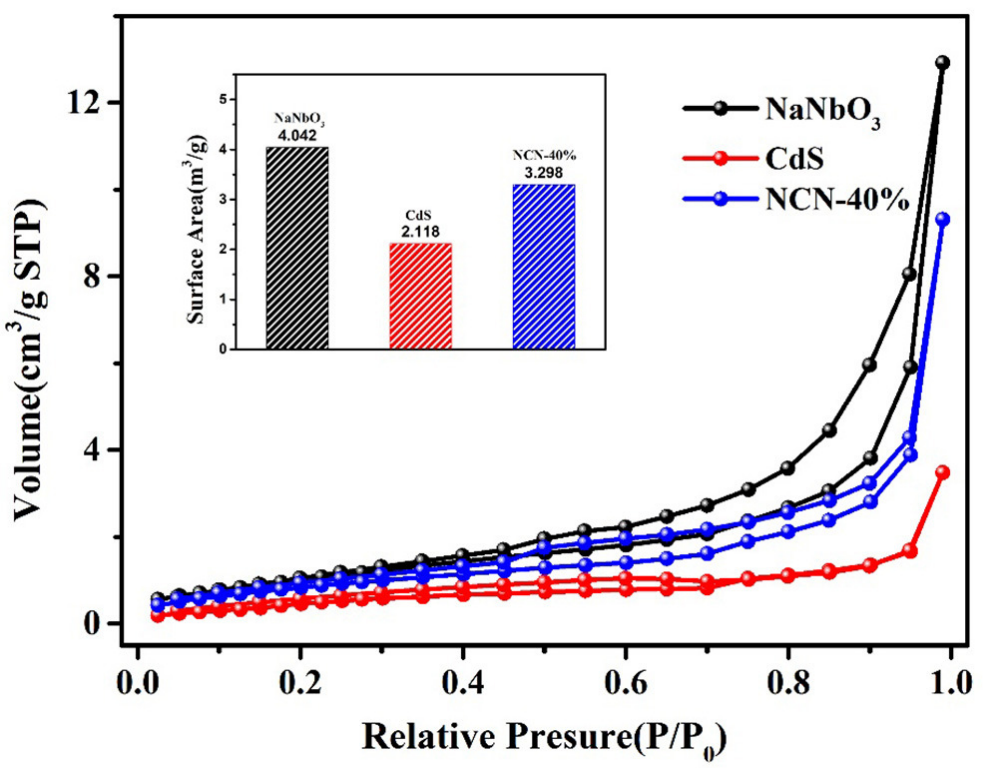
Nitrogen adsorption–desorption isotherms of NaNbO_3_, CdS, and NCN40%. *Inset* BET surface area of NaNbO_3_, CdS, and NCN40%.

The N_2_ adsorption–desorption isotherm was obtained for analysis of the SBET (BET surface area). It could be found in Figure 8 that the isotherm of NaNbO_3_ and CdS was corresponding to type-p and type-n, respectively. The growth of CdS and NiS_2_ on NaNbO_3_ nanocubes improved the SBET of pure CdS, making more contact area with water and light.

**Figure 9:**
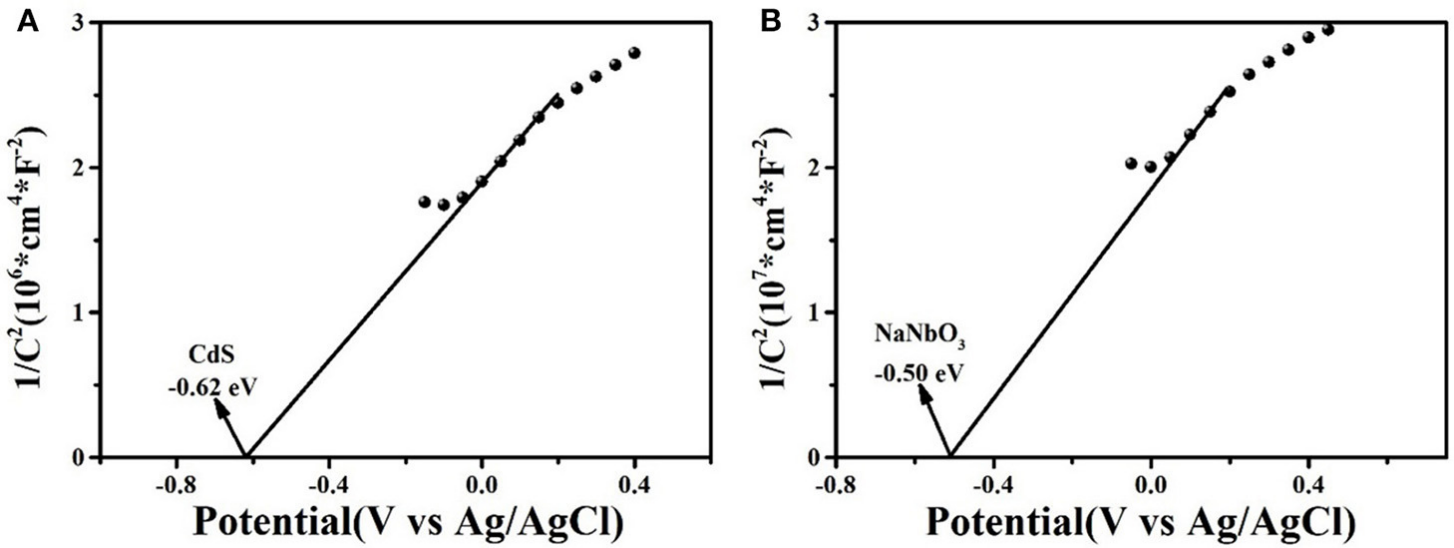
M–S plots of **(A)** CdS and **(B)** NaNbO_3_.

Figure 9 was the Mott–Schottky curves of as-synthesized CdS and NaNbO_3_ samples to confirm the conduction band potentials. According to the positive slope (Guohui et al., 2016; Guo et al., 2019; Zhang et al., 2020), CdS and NaNbO_3_ were both n-type semiconductors. The flat band potentials of CdS and NaNbO_3_ were −0.62 and −0.50 eV, while the normal hydrogen electrode (NHE) could be transformed to −0.52 and −0.40 eV according to equation (*E*_NHE_ = *E*_Ag/Agcl_ + 0.1) (Yue et al., 2019), respectively. Based on the *E*_g_ measured above, the valence band position of CdS and NaNbO_3_ was 1.61 and 2.9 eV, respectively. This staggered energy level position was more favorable for the separation of charges and holes in the heterojunction formed in the composite (Kaowphong et al., 2019).

**Figure 10:**
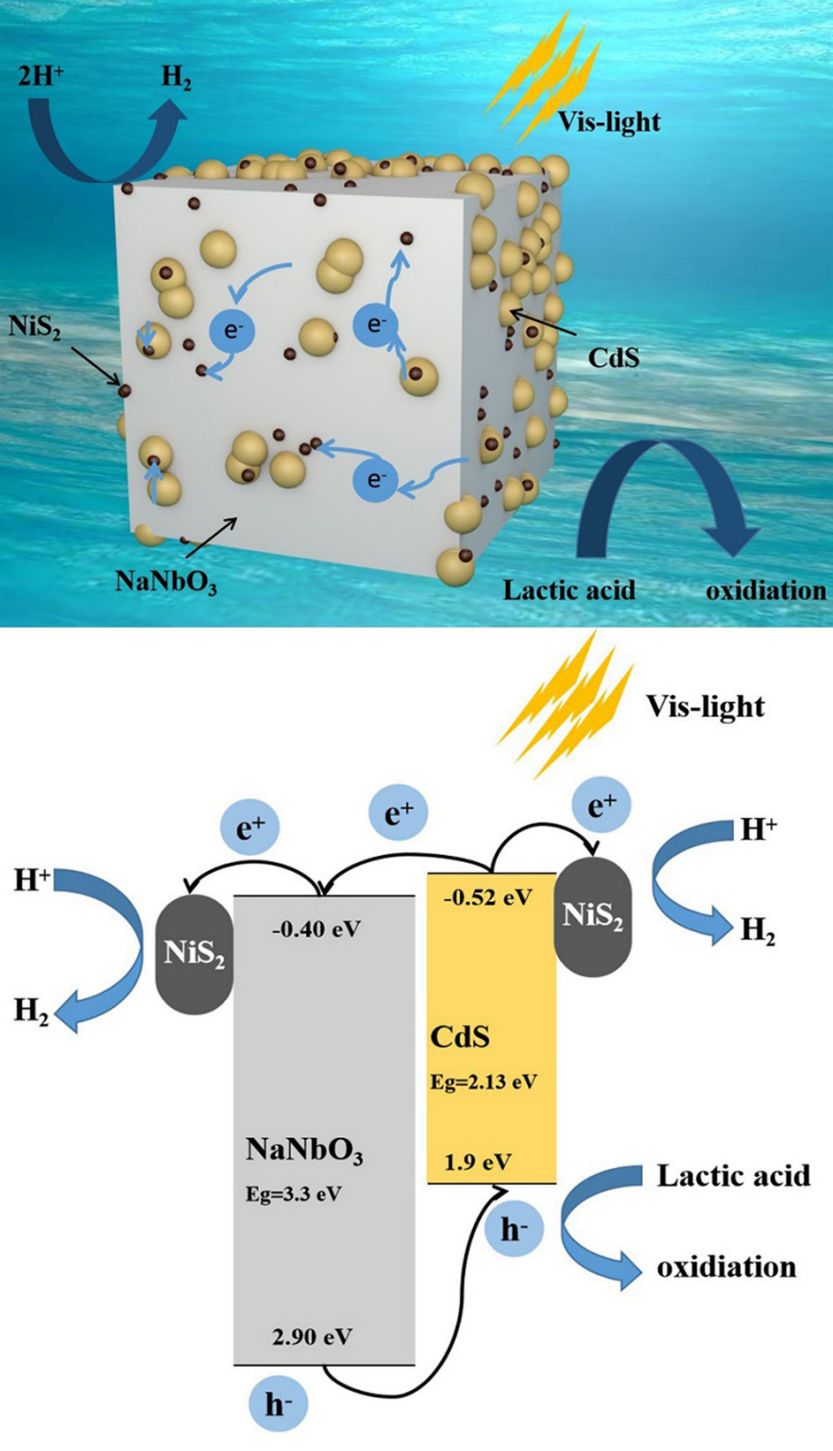
Possible mechanism of photocatalytic H_2_ evolution over NCN40%.

According to the abovementioned research, the possible mechanism of NaNbO_3_/CdS/NiS_2_ ternary catalyst to improve hydrogen evolution efficiency is proposed in Figure 10. Both CdS and NiS_2_ nanoparticles uniformly deposited on NaNbO_3_ nanocubes, which greatly reduced the agglomeration of CdS, thus increasing the active sites for hydrogen evolution. CdS responds to visible light, producing electrons and holes. Then, multi-heterojunctions formed by NaNbO_3_-CdS, CdS–NiS_2_, and NaNbO_3_-NiS_2_ promote shifting electrons from CdS to NaNbO_3_ and NiS_2_, while holes left at CdS are consumed by the sacrificial agent. At the same time, NiS_2_ in contact with NaNbO_3_ or CdS acts as a cocatalyst to further aggregate electrons, providing a number of stable hydrogen production active sites. In general, the synergistic effect of NaNbO_3_ and NiS_2_ can accelerate the migration of electrons and holes, in addition to promoting the hydrogen production efficiency of NaNbO_3_/CdS/NiS_2_ catalytic system.

## Conclusions

In brief, we synthesized noble-metal-free NaNbO_3_/CdS/NiS_2_ ternary composite by two-step hydrothermal synthesis. Under visible light irradiation, H_2_ production rate of ternary catalyst was about 24.7 times that of pure CdS. These may be the possible mechanisms. First, CdS and NiS_2_ nanoparticles were uniformly grown on NaNbO_3_, which greatly increases the light-receiving area and the reaction area with water, and also improved the repeatability of photocatalytic hydrogen evolution. Second, the simultaneous effect of multi-heterojunction structures and active sites facilitated the migration of photogenerated electrons and holes.

## Data Availability Statement

The raw data supporting the conclusions of this manuscript will be made available by the authors, without undue reservation, to any qualified researcher.

## Author Contributions

JX and MC designed the project, guided the study, and polished the manuscript. JZ conducted the experiments and characterized the samples. JY and JN revised the manuscript.

### Conflict of Interest

The authors declare that the research was conducted in the absence of any commercial or financial relationships that could be construed as a potential conflict of interest.
